# Zika virus infection in neonatal mice disrupts connexin 43 and induces cardiac inflammation, implicating viral myocarditis in neonatal pathogenesis

**DOI:** 10.1128/jvi.00871-25

**Published:** 2025-08-05

**Authors:** Shuxuan Li, Wangheng Hou, Najealicka Armstrong, Huan Zhao, Ruth Cruz-Cosme, Hongwei Yang, Jianhua Xu, Hongyu Chen, Chunlian Zhong, Wenkun Fu, Wei Wang, Rui Zhu, Ningshao Xia, Tong Cheng, Qiyi Tang

**Affiliations:** 1 School of Medicine, Henan University of Chinese Medicine 663994 , Zhengzhou, P. R. China; 2 State Key Laboratory of Molecular Vaccinology and Molecular Diagnostics, National Institute of Diagnostics and Vaccine Development in Infectious Diseases, School of Public Health, School of Life Sciences, Xiamen University 12466, Xiamen, P. R. China; 3 Department of Microbiology, Howard University College of Medicine 12232, Washington, DC, USA; 4 School of Material and Chemical Engineering, Minjiang University 26465, Fuzhou, P. R. China; St. Jude Children's Research Hospital, Memphis, Tennessee, USA

**Keywords:** Zika virus (ZIKV), neonatal mouse, pathogenesis, heart diseases, flavivirus, inflammation

## Abstract

Zika virus (ZIKV) is primarily transmitted through mosquito bites and, occasionally, via breast milk, making postnatal ZIKV infections common among newborns and infants, particularly in tropical regions. Previous studies, including ours, have demonstrated that neonatal ZIKV infection can be fatal, highlighting a severe health issue of ZIKV in newborns. However, the pathogenesis and functional outcomes of postnatal ZIKV infection remain largely unexplored. The mechanisms underlying organ failure in infected neonates are still unknown. Here, we investigated postnatal ZIKV (PRVABC59) infection in neonatal mice and found significant cardiac abnormalities. Electrocardiogram (EKG) analysis revealed extended P-R intervals (indicative of the atrioventricular block), widened QRS complexes (suggesting intraventricular block), and elevated ST wave (a biomarker of myocardium impairment), implying defects in myocardial conduction. In addition, ZIKV infection caused increased levels of cTnT, cTnI, CK, CK-MB, CCL2, CXCL9, and CXCL10—biomarkers associated with cardiovascular diseases and infarction-like myocardial pathology. To further elucidate the underlying mechanisms, we analyzed cytokine and chemokine responses and observed a significant increase in multiple inflammatory mediators, including M-CSF, LIF, IL-6, IL-15, CCL2, CCL4, CCL5, CCL11, CXCL1, CXCL9, CXCL10, TNF-α, and VEGF. Notably, ZIKV infection also led to the degradation of connexin 43 (Cx43), a critical protein involved in heart development and intercellular communication among myocardial cells. In summary, our neonatal mouse model of ZIKV infection suggests that ZIKV-induced myocarditis and cardiac dysfunction may contribute to fatal outcomes in newborns. These findings provide new insights into ZIKV pathogenesis and underscore the need for further research into its impact on the cardiovascular system in early life.

**IMPORTANCE:**

Zika virus (ZIKV) is a known teratogen responsible for microcephaly in neonates born to mothers infected during pregnancy. Mouse models have been instrumental in elucidating ZIKV pathogenesis; however, most published studies utilize interferon (IFN)-compromised animals, either genetically modified or antibody-treated. In this study, we employed immunocompetent neonatal mice to investigate postnatal ZIKV infection and uncovered its impact on heart function. We detected high viral loads in heart tissue at early, middle, and late stages of infection using RT-qPCR. Electrocardiogram (EKG) analysis demonstrated cardiac dysfunction, including conduction abnormalities. At the same time, elevated levels of cTnT, cTnI, CK, CK-MB, LDH, α-HBDH, CCL2, and CXCL10—hallmarks of cardiovascular pathology—suggested inflammatory responses associated with heart failure. These findings indicate that neonatal mortality following postnatal ZIKV infection may be driven by virus-induced cardiac dysfunction. Our results provide new insights into ZIKV pathogenesis, suggesting that postnatal ZIKV infection poses a significant risk for severe cardiac disease in neonates.

## INTRODUCTION

Zika virus (ZIKV) was first isolated from a monkey in Uganda’s Zika Forest and later from humans in Nigeria (1). Since 2007, ZIKV has caused epidemic outbreaks of different scales in Micronesia, French Polynesia, the Cook Island, and Easter Island, establishing itself as an emerging arbovirus (2, 3). The widespread pandemic in South America, particularly in Brazil, led the World Health Organization (WHO) to declare ZIKV infection a public health emergency on 1 February 2016 due to its associated symptoms and complications (4). ZIKV, together with West Nile virus, Yellow fever virus, Japanese encephalitis virus, Dengue fever virus, and many other viruses, is a member of the genus *Orthoflavivirus* (5) of the family *Flaviviridae* (6–8). A growing number of strains of ZIKV have been isolated from more than 60 countries (9, 10). *In vivo* studies using mouse models revealed that ZIKV can pass through the placental barrier to infect the fetus, and the infected fetus may die or develop microcephaly or other malformations of the brain (11–16). ZIKV infection has been related to the increasing number of cases of microcephaly and Guillain-Barré syndrome (GBS) in the areas of the epidemics (9, 17, 18, 19).

While congenital viral infection has been a primary focus of ZIKV studies, postnatal ZIKV infection has recently garnered attention (20–22). ZIKV is primarily transmitted via mosquito bites and breast milk (23–27), making newborns and infants, particularly in tropical regions, vulnerable to postnatal infection. Until recently, the potential for severe clinical consequences from postnatal ZIKV infection remained unclear. However, *in vivo* studies using mice and monkeys have begun to shed light on this issue (28–30). Wild-type (WT) mice used in ZIKV infection studies have predominantly been neonates (22, 31, 32). One-day-old WT C57BL/6 mice were shown to be susceptible to ZIKV infection, developing symptoms post-infection (32). Despite these findings, the pathogenesis and functional outcomes of postnatal ZIKV infection remain understudied. We recently systematically investigated ZIKV infection in neonatal mice, examining pathogenesis, virus-host interaction, and neuronal damage (22). Our findings, along with those of other groups, demonstrated that ZIKV not only replicates in WT neonatal mice but also causes fatal outcomes (15, 16, 22, 31–34). Notably, ZIKV infection in 1-day-old or 3-day-old mice resulted in 100% mortality (22). However, the mechanisms underlying neonatal death due to ZIKV infection remain unknown. Beyond its well-documented effects on fetal brain development, ZIKV has also been implicated in heart disease (35, 36). A case of myocarditis was reported in an adult traveler infected with ZIKV (37), raising the question of whether ZIKV infection could also lead to neonatal heart failure.

ZIKV infection has been reported to trigger cytokine release syndrome (CRS), also known as a cytokine storm, leading to the activation of proinflammatory cytokines and monocyte proliferation (38). The excessive production of cytokines and chemokines facilitates the recruitment of immune cells to the site of infection, aiming to control viral spread and initiate an adaptive immune response. However, this immune response can also result in tissue damage at sites of immune cell aggregation. Additionally, a clinical study reported elevated levels of various cytokines in patients with Zika fever (39). In general, viral-induced inflammation is a well-established cause of myocarditis and other heart diseases (40, 41). Furthermore, ZIKV has been detected in multiple organs through PCR and immune-fluorescence assays (22), raising the question of whether it contributes to heart diseases in neonatal mice following postnatal infection. To investigate this possibility, we infected neonatal mice with ZIKV and found that ZIKV infection is associated with heart failure.

## MATERIALS AND METHODS

### Mice

To investigate viral infection and pathogenesis in neonatal mice, pregnant C57BL/6 mice were purchased from the SLAC Laboratory Animal Center (China) or from Jackson Laboratory (JAX, Bar Harbor, ME, USA). The mice were housed under specific pathogen-free conditions with free access to food and water in sterile micro-isolator cages. All neonatal mice used in this study were born in-house, and 1-day-old newborns from multiple litters were maintained with the dam until use. For the isolation of murine cardiomyocytes, 4-week-old C57BL/6 (the Jackson Laboratory, Stock #000664) were purchased and bred in-house to generate 1-day-old mice from which cardiomyocytes were isolated. For the experiments, different numbers of mice were used, and all the experiments have been performed for three times independently.

### Cells and viruses

Vero cells and primary cardiomyocytes were used in this study. Vero cells (African green monkey kidney epithelial cells) were maintained in DMEM supplemented with 10% fetal bovine serum (FBS, Gibco), 100 U/mL of penicillin, 100 µg/mL of streptomycin, and 2 mM L-glutamine at 37°C with 5% CO_2_. Primary cardiomyocytes used in this study were isolated from 1-day-old neonatal mice. Briefly, 1-day-old C57BL/6 mice were euthanized, and hearts were isolated. Cardiomyocytes (42) were then isolated using the Primary Cardiomyocyte Isolation Kit (#88281, ThermoFisher Scientific, USA) according to the manufacturer’s protocol. The clinical isolate of Asian lineage ZIKV strain PRVABC59 (GenBank accession number KU501215, Puerto Rico, 2015) (43) and ZIKV strain MR766 were purchased from ATCC and used in this study. The viruses were passaged three times in Vero cells in our laboratory to generate virus stock (44). The virus titer was determined in Vero cells and expressed as 50% tissue culture infectious dose (TCID_50_) using the Reed and Muench method as in our previous work (45, 46).

### Animal experiments

For viral infection studies, 1-day-old C57BL/6 mice were inoculated intraperitoneally with PRVABC59 or MR766 at a dose of 10^4^ TCID_50_ per mouse in 10 µL, while control mice received 10 µL of PBS per mouse. Mice were monitored daily for clinical signs of pathology and weighed every day as described in our previous study (27). For the serum collection, blood was obtained via cardiac puncture under anesthesia. After incubation at 37°C for 30 min, the samples were centrifuged at 12,000 rpm for 10 min to harvest serum, which is subsequently used for the viral RNA load detection and cytokine assays. For tissue sampling, the heart and spleen were aseptically removed and weighed at 6 and 11 dpi. Tissues were stored at −80°C until further analysis.

### Quantitative real-time PCR analysis

Real-time PCR was utilized to quantify viral RNA levels in serum and heart tissues, as well as to assess the relative expression of the Cx43 gene in cardiomyocytes. Prior to RNA extraction, harvested heart tissues were briefly rinsed in PBS to remove residual blood by pumping for 1–3 min. The tissues were then weighed and homogenized in 500 µL PBS using a mechanical homogenizer (Scientz-192, Scientz, China). Total RNA was extracted from serum and heart tissue homogenates using the GenMagSpin Viral DNA/RNA Kit (GenMag Biotech, China), while RNA from cardiomyocytes was isolated using TRIzol reagent (Invitrogen, USA). A list of primer sequences utilized in this study is provided in Table 1. To determine viral RNA loads, total RNA was reverse-transcribed into cDNA and subjected to real-time PCR using a One-Step RT-PCR Kit (GenMag Biotech, China) following the manufacturer’s protocol. The reaction conditions were set as follows: 42°C for 15 min and 95°C for 15 min, followed by 40 cycles of 95°C for 15 s and 55°C for 55 s. A standard curve was generated to quantify RNA copy numbers. Specifically, a ZIKV fragment (primer sequence in Table 1) was amplified and cloned into the pMD18-T Vector to generate the recombinant plasmid pMD18-ZIKV, which served as the standard control. The copy numbers of the standard sample were determined using an online website (http://cels.uri.edu/gsc/cndna.html). Serially diluted standard samples were used to construct a standard curve, which was applied in each PCR to quantify viral RNA levels in serum and heart tissues. The detection threshold was set at 10^3^ copies/mL of serum or per milligram of tissue. For the determination of relative Cx43 gene expression, total RNA from cardiomyocytes was reverse-transcribed into cDNA using the First-Strand cDNA Synthesis Kit (Invitrogen). The reverse transcription reaction was carried out at 42°C for 30 min, followed by a denaturation step at 95°C for 15 min, and the resulting cDNA products were stored at −20°C until further analysis. Quantitative real-time PCR was performed using the SYBR Green PCR Kit (ThermoFisher Scientific, USA) following the manufacturer’s protocol. The PCR conditions were pre-denaturation at 95°C for 5 min, followed by 40 cycles of denaturation at 95°C for 30 s, annealing at 60°C for 30 s, and extension at 72°C for 30 s. The relative quantification of the Cx43 gene was normalized to GAPDH as the internal reference gene, and the fold change in expression was calculated using the 2^−ΔΔCT^ method.

**TABLE 1:** Sequences of primers used in this study

Name	Primer	Sequence (5′−3′)
ZIKV	ZIKV-forward	TGTCTGACAAAGGCTGGAAA
	ZIKV-reverse	AYGACRAAGTCCCACTCTTGAT
	ZIKV-probe	ROX-ATACAGCTCAGCAGRAAGACTTTTGAGA-BHQ2
Cx43	Cx43-forward	CCCCACTCTCACCTCTGTCTCC
	Cx43-reverse	ACTTTTGCCGCCTAGCTATCCC
GAPDH	GAPDH-forward	CATCACTGCCACCCAGAAGACTG
	GAPDH-reverse	ATGCCAGTGAGCTTCCCGTTCAG

### Electrocardiographic assessment

To evaluate the cardiac function of ZIKV-infected mice, EKG recordings were performed at 11 dpi. Mice (*n* = 6 per group) were anesthetized using sodium pentobarbital, and four electrodes were inserted subcutaneously into the limbs. EKG signals were recorded for approximately 10 min at a sample rate of 1 KHz using a direct writing oscillograph (BL420S, Chengdu Techman Software Co., Ltd., China). Representative 450 ms segments of high-quality signals are shown in this study.

### Cytokine and chemokine detection assay

Serum samples collected from ZIKV- or PBS-infected mice at 11 dpi were analyzed for cytokine expression using the Mouse Cytokine Array Panel A Kit (#ARY006, R&D Systems, USA) (*n* = 3 per group), which detects 40 different murine cytokines, following the manufacturer’s protocol. Data were analyzed using Image J Software and presented as mean pixel density. Additionally, a quantitative cytokine assay was performed to assess cytokine and chemokine levels in both serum and heart tissues from neonatal mice infected with ZIKV or PBS at 6 and 11 dpi (*n* = 3–5 per group). The Milliplex map mouse Cytokine/Chemokine Magnetic Bead Panel Kit (#MCYTMAG-70K-PX32, Merck Millipore, USA) was used to quantify the following biomarkers via Luminex technology: G-CSF, M-CSF, GM-CSF, LIF, LIX, IL-1α, IL-1β, IL-2, IL-3, IL-4, IL-5, IL-6, IL-7, IL-9, IL-10, IL-12(P40), IL-12(P70), IL-13, IL-15, IL-17, CCL2, CCL3, CCL4, CCL5, CCL11, CXCL1, CXCL2, CXCL9, CXCL10, IFN-γ, TNF-α, and VEGF. The plates were read immediately using a Luminex instrument with xPONENT Software for data acquisition and analysis.

### Detection of cardiac injury markers

Serum and heart tissue samples collected from ZIKV-infected or PBS-treated mice at 6 and 12 dpi (*n* = 8 per group). Quantitative assays of levels of cTnT, cTnI, and CK-MB in the serum and heart tissue samples were measured by UMIC Wan200+ Fully Automated Chemiluminescence Immunoassay Analyzer according to the manufacturer’s protocols (#20142400052, #XS 20232400158, #20152400132; XIAMEN INNODX BIOTECHNOLOGY CO LTD, China). Their concentrations were shown in μg/L. The levels of LDH, α-HBDH, and CK were measured by UMIC BC2000 Fully Automated Biochemical Analyzer according to the manufacturer’s protocols (#XS0370, #XS0470, #XS0170; BEIJING WANTAI DRD CO., LTD, China) and presented as U/L.

### Immunohistochemical staining

Following anesthesia, heart tissues were collected, fixed in 4% formalin (in PBS), embedded in paraffin, and sectioned into 4 μm-thick slices. The sections were deparaffinized, rehydrated, and treated with 3% H_2_O_2_ (in methanol) to block endogenous peroxidase activity. After antigen retrieval, IHC staining was performed using an Ultrasensitive TM S-P Kit (Maixin Biotech, China) and the DAB Detection Kit (Streptavidin-Biotin; Maixin Biotech, China) following the manufacturer’s protocols. The primary antibodies used for staining include anti-Cx43 (Sigma, cat# C6219 1:2,000), anti-ZIKV NS3 protein antibody (monoclonal antibody, 7A9, generated in our lab, 1:200), and anti-Cleaved Caspase-3 antibody (Cell Signaling Technology, cat# 9661, 1:200). Positive staining was indicated by a brown color in the tissue sections.

### Masson staining

Masson’s trichrome staining was performed to assess myocardial fibrosis. Paraffin-embedded heart tissue sections were deparaffinized, rehydrated, and stained with Masson’s Trichrome staining Kit (#MST-8004, Maixin Biotech, China) following the manufacturer’s instructions.

### Western blotting

Western blotting was performed to assess the expression of Cx43, Tubulin, and ZIKV NS3 proteins in the heart tissues homogenized from mice or primary cardiomyocytes subjected to different treatments. Heart tissues were homogenized, and cells were lysed in RIPA buffer (#P0013B, Beyotime, China) supplemented with proteinase inhibitors (#P1005, Beyotime, China) on ice. Protein concentration was determined using a bicinchoninic acid (BCA) Protein Assay Kit (#23225, ThermoFisher Scientific, USA). Protein samples were denatured in loading buffer, separated via 10% SDS-PAGE, and transferred onto a nitrocellulose membrane (Whatman). The membranes were blocked by 5% nonfat milk in PBS for 30 min at room temperature, followed by overnight incubation at 4°C with primary antibodies, including anti-ZIKV NS3 antibody (a monoclonal antibody, 7A9, produced in our lab, 1:1,000), anti-Cx43 antibody (Sigma, cat# C6219, 1:5,000), and anti-Tubulin antibody (Santa Cruz, CA, 4G1, sc-58666). After washing five times with PBS containing 0.1% Tween 20 (PBST), the membranes were incubated with HRP-conjugated secondary antibodies for 1 h at room temperature. The protein signals were visualized using the Pierce ECL Western blotting Substrate (#34095, ThermoFisher Scientific, USA).

### Statistical analyses

All statistical analyses were performed using GraphPad Prism 8.3.0 (GraphPad Software, USA). Comparisons between two groups were conducted using the Student’s *t*-test. A *P* value < 0.05 was considered statistically significant. Data were shown as mean ± SEM, as indicated.

## RESULTS

### ZIKV infection in neonatal mice leads to viral replication in the heart and induces cardiac enlargement

**Fig 1:**
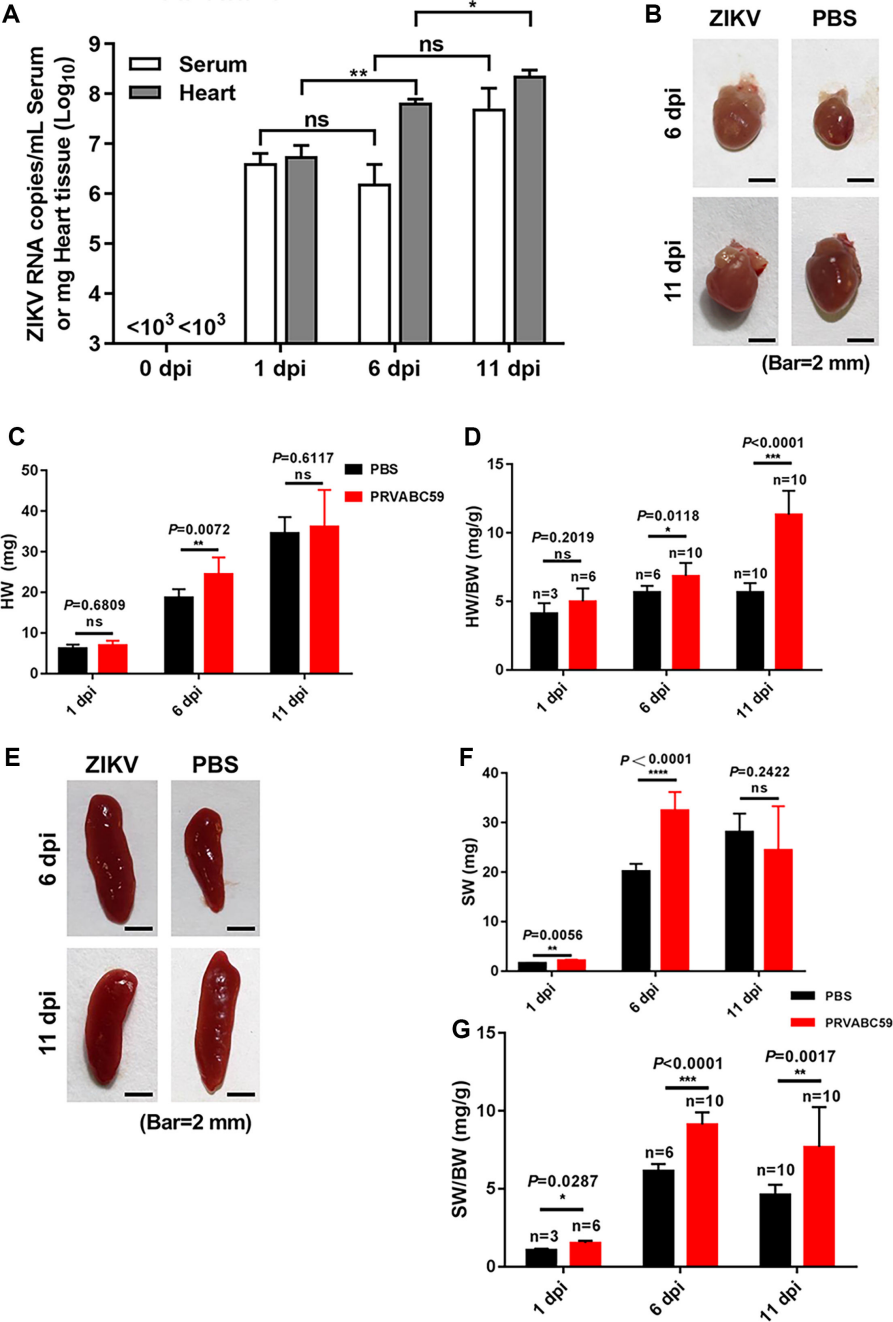
ZIKV replication, heart enlargement, and spleen alterations in neonatal mice. One-day-old C57BL/6 mice were injected intraperitoneally with the ZIKV strain PRVABC59 (10⁶ TCID₅₀/mouse) or PBS as a control, and serum and tissue samples were collected at 0, 1, 6, and 11 dpi. (**A**) ZIKV RNA levels in serum and heart tissue were quantified by real-time RT-PCR using ZIKV-specific primers (*n* = 3/group). (**B**) Representative images of whole hearts from ZIKV-injected and PBS-treated mice at 6 and 11 dpi. Scale bars, 2 mm. (**C and D**) Absolute heart weight (**C**) and heart-to-body weight ratio (HW/BW) (**D**) of ZIKV-infected and PBS-treated mice at 1, 6, and 11 dpi (*n* = 3–10 per group). Error bars indicate SEM. (E–G) Effects of ZIKV infection on spleen morphology and weight. (**E**) Representative images of whole spleens from ZIKV-infected and PBS-treated mice at 6 and 11 dpi. Scale bars, 2 mm. (**F, G**) Spleen-to-body weight ratio (SW/BW) (**F**) and absolute spleen weight (**G**) in ZIKV-infected and PBS-treated mice at 1, 6, and 11 dpi (*n* = 3–10 per group). Statistical significance: ns, *P* > 0.05; *P < 0.05; **P < 0.01; ***P < 0.001; ****P < 0.0001.

A high incidence of cardiac defects has been observed in infants congenitally infected with ZIKV (47). While previous studies have linked postnatal ZIKV infection to heart disease (35), the effects of neonatal ZIKV infection remain largely understudied, with limited reports on postnatal ZIKV infection-associated heart diseases. Therefore, experimental studies in an animal model are crucial to elucidate the causal relationship between ZIKV infection and myocardial damage. To determine whether ZIKV contributes to heart disease, we first examined ZIKV replication in the heart following infection. One-day-old mice were infected with PRVABC59 at a dose of 10^4^ TCID_50_ per mouse. Heart tissues and blood samples were collected at 0, 1, 6, and 11 dpi (days post-infection). The hearts were washed and homogenized, and total RNA was extracted for quantification of viral RNA copies via RT-qPCR. Primer sequences are provided in Table 1. As shown in Fig. 1A, ZIKV replication was detected early after viral infection, with a marked increase in viral RNA copies in both heart tissue and blood plasma at 1 dpi, which persisted at higher levels through 6 and 11 dpi. Notably, at 6 and 11 dpi, viral RNA levels in the heart surpassed those in the blood plasma, indicating sustained viral replication in cardiac tissue.

Next, we monitored heart development in neonatal mice over 11 days after birth post-infection. As shown in Fig. 1B and C, hearts from the ZIKV-infected mice were significantly larger than those from non-infected mice at 6 dpi though no significant differences were observed at 11 dpi. However, given the developmental defects and reduced body weights in ZIKV-infected neonatal mice (22), the heart weight-to-body weight (HW/BW) ratio was significantly higher in ZIKV-infected mice compared to the non-infected ones (Fig. 1D) at 11 dpi. Similarly, we evaluated spleen development in neonatal mice over 11 days post-birth. As presented in Fig. 1E and F, spleens from ZIKV-infected mice were significantly larger than those from non-infected mice at 6 dpi, with no significant differences at 11 dpi. However, the spleen weight-to-body weight (SW/BW) ratio remained significantly elevated in infected mice at both 6 and 11 dpi (Fig. 1G). Collectively, these findings demonstrate that ZIKV not only replicates in the hearts of neonatal mice but also induces significant cardiac enlargement, suggesting a potential link between postnatal ZIKV infection and cardiac pathology.

### ZIKV infection induces abnormal myocardial function in neonatal mice as assessed by EKG

**Fig 2:**
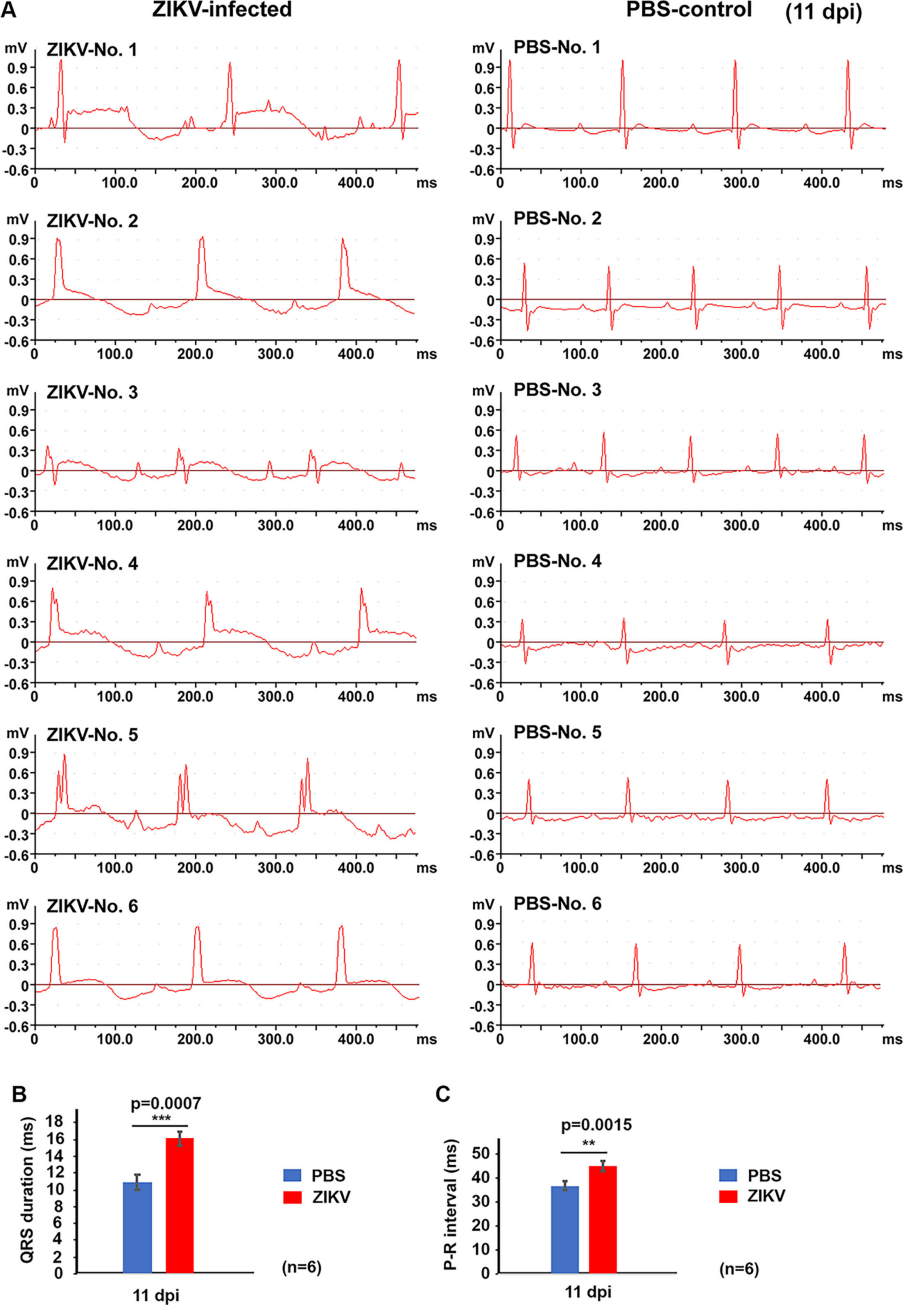
Electrocardiogram (EKG) abnormalities in neonatal mice following ZIKV infection. (**A**) One-day-old C57BL/6 mice were injected intraperitoneally with ZIKV strain PRVABC59 and were recorded for approximately 10 min using a direct-writing oscillograph. Representative 450 ms segments of high-quality EKG tracings were shown for ZIKV-infected (left) and PBS-treated (right) mice. (**B**) Representative EKG tracings of neonatal mice at 11 dpi, comparing ZIKV-infected and PBS-treated controls. (**C**) QRS duration in ZIKV-infected vs PBS-treated mice (*n* = 3–7 per group). ***P < 0.001.

To determine whether ZIKV infection leads to functional abnormalities in the heart, we performed electrocardiogram (EKG) recordings on six mice per group (PBS-treated vs ZIKV-infected) at 11 dpi and statistically analyzed the QRS durations. Representative EKG patterns from all six mice in each group are shown in Fig. 2A. As can be seen in Fig. 2A, PBS-treated mice showed normal EKG patterns at 11 dpi, characterized by regular heartbeats, normal P-R intervals, QRS complexes, S-T intervals, and standard T waves—indicative of normal cardiac function. In contrast, ZIKV-infected mice displayed abnormal EKG patterns, including a slower heartbeat, widened P-R intervals, broadened QRS complexes, and elevated ST segments, all suggesting myocardial injury associated with ischemia and hypoxia. Specifically, prolonged P-R intervals are indicative of atrioventricular block, widened QRS complexes suggest intraventricular conduction delay, and elevated ST segments reflect myocardial impairment.

Quantitative analysis revealed a significant widening of the QRS duration in ZIKV-infected mice compared to PBS-treated controls at 11 dpi (Fig. 2B). Similarly, P-R intervals were significantly prolonged following ZIKV infection (Fig. 2C). These findings suggest that ZIKV infection leads to myocardial dysfunction, likely due to virus-induced cardiac pathology.

### ZIKV infection leads to elevated cytokine and chemokine levels in neonatal mice

Although the observed EKG abnormalities suggest that ZIKV infection contributes to heart disease, the underlying mechanisms driving ZIKV-induced cardiac dysfunction remain unknown. Viral infections are typically associated with inflammatory responses, which may contribute to cardiac pathology. Consistent with our previous study, hematoxylin and eosin (H&E) staining of heart tissue from ZIKV-infected mice revealed extensive vacuolar degeneration accompanied by inflammatory infiltration (22). To determine whether these cardiac abnormalities are linked to ZIKV-induced inflammation, we examined the effects of ZIKV infection on innate immune responses, specifically cytokine and chemokine production, in neonatal mice.

**Fig 3:**
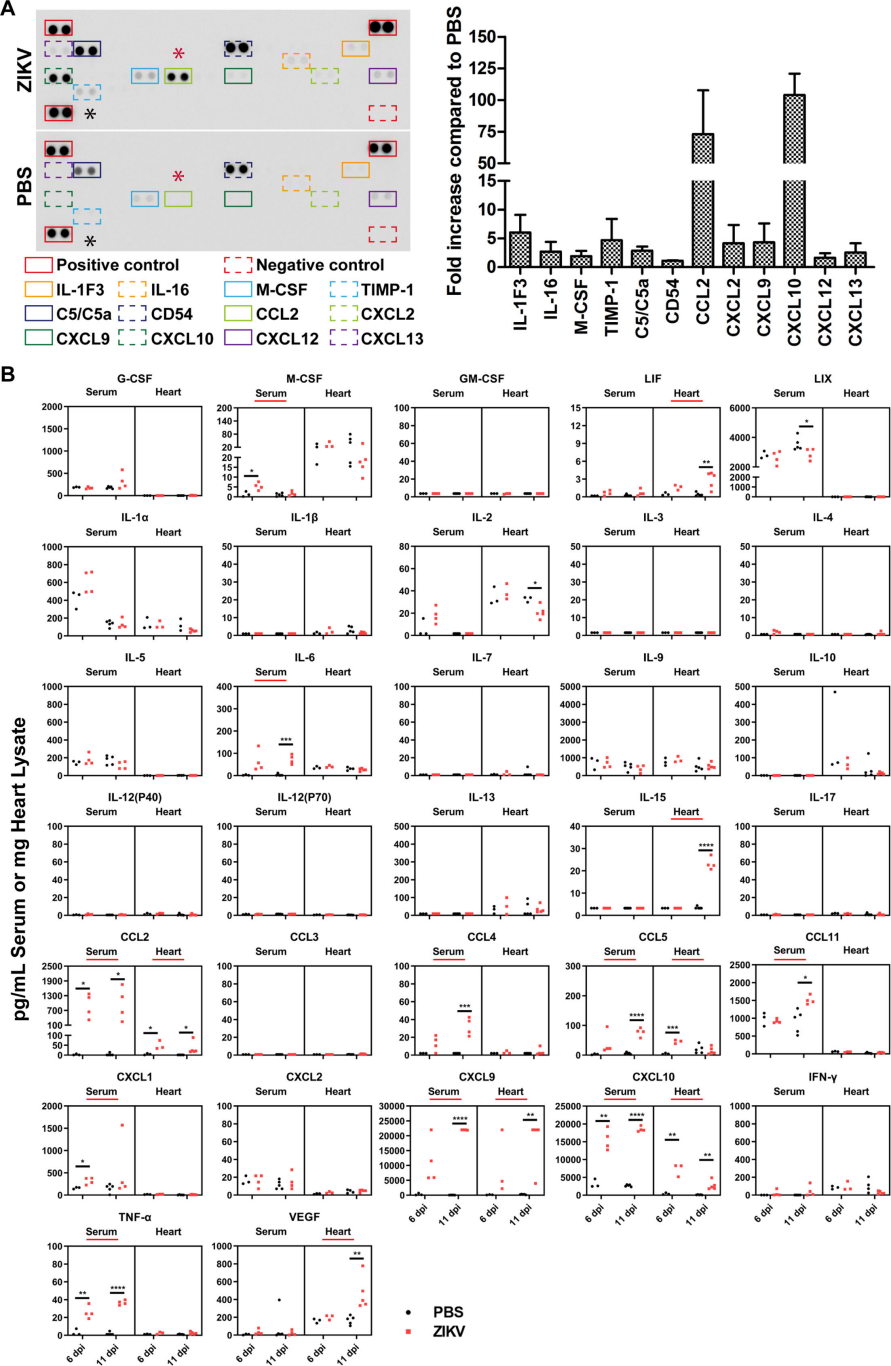
Expression levels of cytokines and chemokines in neonatal mice following ZIKV infection. One-day-old C57BL/6 mice were injected intraperitoneally with PRVABC59 (10^6^ TCID_50_/mouse) or PBS. (**A**) Protein array analysis and quantification. Levels of 40 different cytokines and chemokines were measured in serum samples collected at 11 dpi using Mouse Cytokine Array (*n* = 3 per group). Left panel: a representative image from triplicate samples is shown, with elevated cytokines and chemokines highlighted by squares and labeled below. The most elevated cytokines, CCL2 and CXCL10, were indicated by an asterisk. Right panel: Data from triplicates were quantified by Image J Software, normalized to positive and negative controls, and presented as a bar graph. (**B**) Cytokine/chemokine quantification assay. Serum and heart tissue samples were collected at 6 and 11 dpi (*n* = 3-5 per group), and the expression levels of 32 cytokines and chemokines were measured using Luminex technology and analyzed by xPONENT Software. Upregulated cytokines or chemokines are underlined in red. Statistical significance: *P < 0.05; **P < 0.01; ***P < 0.001; ****P < 0.0001.

One-day-old mice were intraperitoneally injected with 10^4^ TCID50 PRVABC59 per mouse, while control mice received an equal volume of PBS. Serum and heart tissue samples were collected at 6 and 11 dpi for analysis. To identify inflammatory factors altered by ZIKV infection, we performed a cytokine array using serum samples collected at 11 dpi from three mice per group. A representative array is shown in the left panel of Fig. 3A, with significantly upregulated cytokines and chemokines listed below. Triplicate experimental data were quantified using ImageJ software, and the normalized average values, relative to positive and negative controls, are presented in the right panel of Fig. 3A. Notably, ZIKV infection led to a 72-fold and 104-fold increase in CCL2 (marked with a red asterisk in 3A) and CXCL10 (notified by a black asterisk in 3A) levels, respectively. Other inflammatory mediators, including IL-1F3, TIMP-1, CXCL2, and CXCL9, were also elevated, albeit at lower levels, and were undetectable in the PBS-treated group.

To further validate these findings, we performed a Luminex immunoassay to quantify multiple cytokines or chemokines (Fig. 3B). ZIKV infection resulted in a significant upregulation of several inflammatory mediators. In the serum, increased levels of M-CSF, IL-6, CCL2, CCL4, CCL5, CCL11, CXCL1, CXCL9, CXCL10, and TNF-α were detected. In the heart tissue, LIF, IL-15, CCL2, CCL5, CXCL9, CXCL10, and VEGF were upregulated (cytokines or chemokines with significant increases are highlighted in red in Fig. 3B). Importantly, CCL2, CCL5, CXCL9, and CXCL10 were consistently elevated in both serum and heart tissue, indicating a systemic and localized inflammatory response induced by ZIKV infection. We summarized the significantly elevated cytokines and chemokines in serum and in heart after ZIKV infection into Table 2.

**TABLE 2:** ZIKV-induced elevations of cytokines or chemokines in serum and in heart

Source	Elevated cytokines/chemokines
Serum	M-CSF, IL-6, CCL2, CCL4, CCL5, CCL11, CXCL1, CXCL9, CXCL10, and TNF-α
Heart	LIF, IL-15, CCL2, CCL5, CXCL9, CXCL10, and VEGF

Notably, CCL2 and CXCL10 are established biomarkers of heart failure and left ventricular dysfunction (48–52), aligning with the observed ZIKV-induced cardiac pathology. These findings raise the question of whether a direct pathophysiological relationship exists between ZIKV-induced chemokine production and the development of heart failure, warranting further investigation.

### ZIKV infection resulted in the upregulation of biomarkers of acute myocardial injury in blood and heart tissues in neonatal mice

To assess ZIKV-induced cardiac injury, we quantified the levels of well-established biomarkers of acute myocardial damage in the blood and heart tissues of neonatal mice. These markers include creatine kinase (CK), the cardiac-specific isoenzyme CK-MB, lactate dehydrogenase (LDH), cardiac troponins T and I (cTnT and cTnI), and alpha-hydroxybutyrate dehydrogenase (α-HBDH) (53–56). PBS-treated mice served as negative controls. Each experimental group (ZIKV-infected and control) consisted of eight mice per time point.

**Fig 4:**
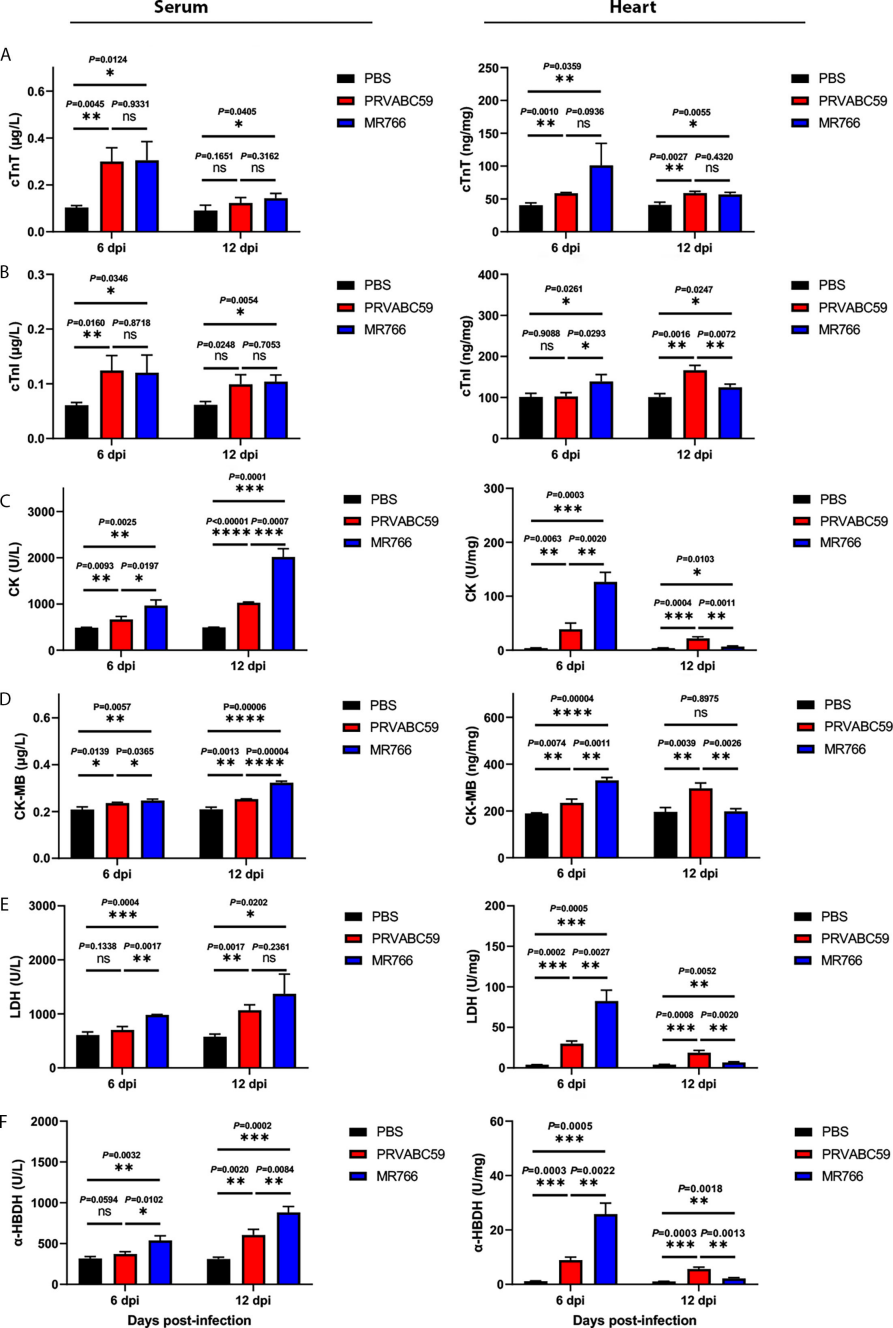
Levels of cardiac muscle enzyme in blood and heart tissues. Three-day-old C57BL/6 mice were injected intraperitoneally with MR766 and PRVABC59 (10^4^ TCID50/mouse) or PBS. Serum and heart tissue samples were collected at 6 and 12 dpi (*n* = 8 per group) for cardiac muscle enzyme detection. (**A, B, F**) The concentrations of cTnT, cTnI, and CK-MB in the serum and heart tissue samples were measured by a UMIC Wan200+ analyzer according to the manufacturer’s protocols. (C through E) Levels of LDH, α-HBDH, and CK were measured by a UMIC BC2000 analyzer according to the manufacturer’s protocols. Statistical significance: *P < 0.05; **P < 0.01; ***P < 0.001; ****P < 0.0001.

As shown in Fig. 4A through F, ZIKV infection resulted in a significant elevation of all six biomarkers in both serum (left panels) and heart tissue (right panels) at 6 days post-infection (dpi). In the blood samples, these markers, typically released into the blood circulation following myocardial cell damage, remained elevated in serum through 12 dpi. In heart tissue, the protein levels of cTnT, cTnI, and CK-MB remained significantly elevated at 12 dpi, comparable to their levels at 6 dpi. In contrast, LDH, α-HBDH, and total CK showed a modest decline by 12 dpi though levels remained higher than those in controls. These findings collectively indicate that ZIKV infection induces sustained cardiac muscle damage, as evidenced by persistent elevation of canonical biomarkers of myocardial injury. Interestingly, we observed significant differences in biomarker elevations between PRVABC59- and MR766-infected mice, highlighting the need for further investigation into pathogenic differences between the two ZIKV clades in neonatal models.

### ZIKV infection disrupts connexin 43 localization and induces myocardial injury in neonatal mice

ZIKV-infected neonatal mice exhibited abnormal electrocardiogram (EKG) profiles, including prolonged P-R intervals and elevated S-T segments (Fig. 2), which are indicative of atrioventricular conduction delay and myocardial injury, respectively. Gap junctions, composed primarily of connexins, are essential for synchronized cardiomyocyte contraction, with connexin 43 (Cx43) being the predominant isoform in ventricular myocytes (57). Disorganization or downregulation of Cx43 has been implicated in impaired electrical conduction and the onset of arrhythmias (58, 59).

**Fig 5:**
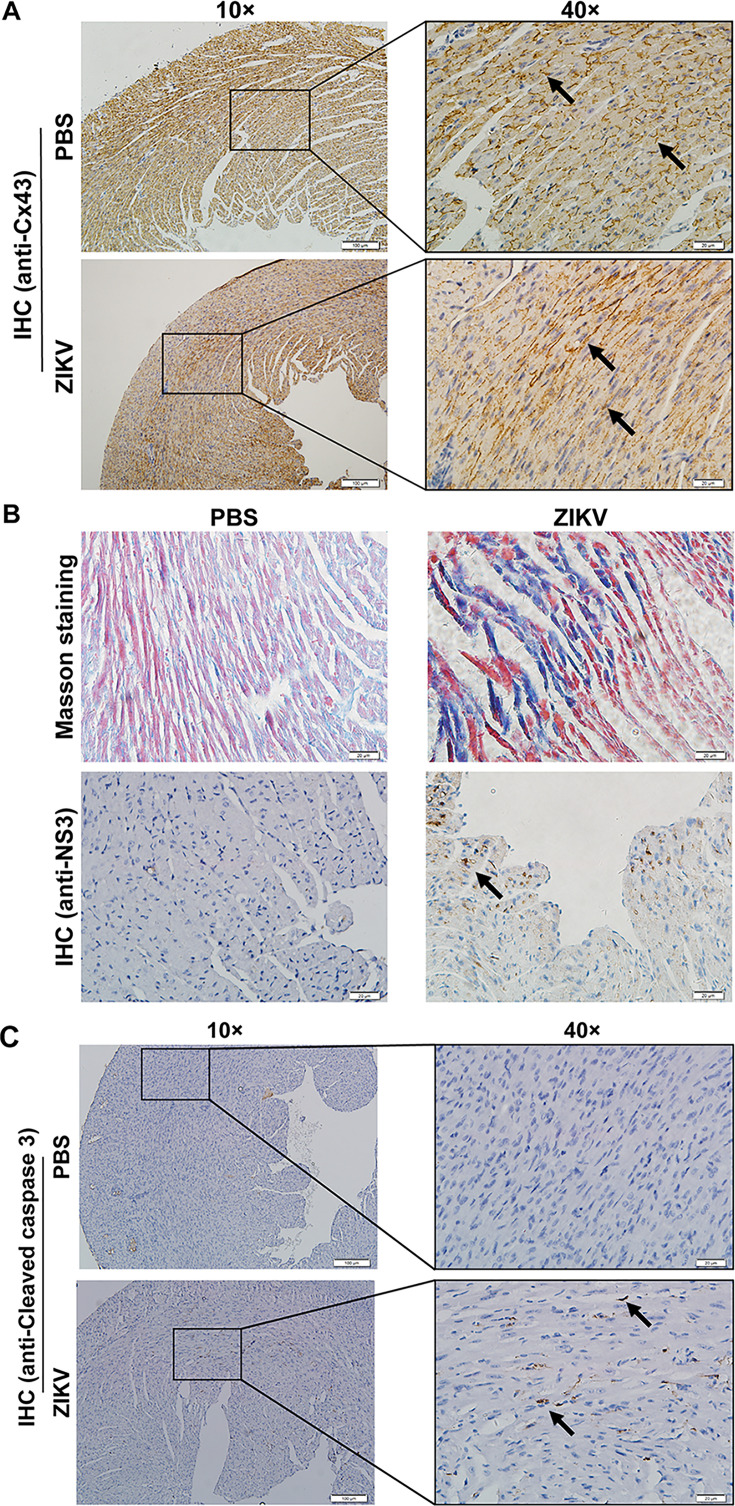
Pathological changes in the heart tissues of ZIKV-infected neonatal mice. Heart tissues were collected at 11 dpi from 1-day-old C57BL/6 mice injected intraperitoneally with ZIKV strain PRVABC59 (10^6^ TCID_50_/mouse) or PBS. (**A**) Immunohistochemical (IHC) staining of Cx43 protein. Representative images of heart tissue cross-sections stained for Cx43 (brown). Scale bars: 100 µm on the left side and 20 µm on the right side. (**B**) Masson’s trichrome staining and IHC analysis of ZIKV protein. Upper panel: Masson trichrome staining highlights cardiac fibrosis (blue). Lower panel: IHC detection of ZIKV protein using anti-NS3 monoclonal antibody (7A9), with positive signals appearing brown. Scale bars: 100 µm (upper) and 20 µm (lower). (**C**) IHC staining of cleaved caspase-3 in the heart tissue. Representative images of heart tissue sections stained with anti-cleaved caspase-3 antibody. Positive apoptotic signals appear brown. Scale bars: 100 µm (left) and 20 µm (right).

To determine whether ZIKV infection affects Cx43 expression and localization, we performed immunohistochemistry (IHC) on heart tissue sections from neonatal mice infected with ZIKV or treated with PBS. In control mice, Cx43 was concentrated in organized linear streaks along the intercalated discs (ICDs), reflecting normal cell–cell coupling (Fig. 5A). In contrast, ZIKV-infected hearts displayed diffuse, irregular Cx43 staining, indicating disruption of gap junction architecture (arrows, Fig. 5A).

To assess myocardial structure, we conducted Masson’s trichrome staining. PBS-treated hearts exhibited normal muscle architecture with minimal fibrosis. ZIKV-infected hearts showed myocyte swelling, disarrayed tissue architecture, and increased fibrotic regions (Fig. 5B), consistent with necrosis and tissue remodeling. Apoptosis was further confirmed by IHC staining for cleaved caspase-3, which was markedly elevated in ZIKV-infected hearts but absent in controls (Fig. 5C). Viral replication in heart tissue was validated using an anti-NS3 antibody (Fig. 5B, bottom panel).

Together, these results indicate that ZIKV infection compromises cardiac electrical connectivity by disrupting Cx43 localization and inducing inflammation, fibrosis, and apoptosis in neonatal hearts. These structural and molecular alterations likely underlie the cardiac dysfunction observed in ZIKV-infected neonates.

### ZIKV infection reduces Cx43 protein levels in neonatal hearts and cardiomyocytes via proteasomal degradation

**Fig 6:**
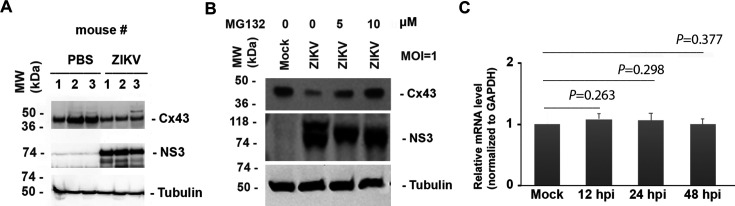
ZIKV infection reduces Cx43 protein levels in neonatal heart tissue and isolated cardiomyocytes. (**A**) Western blotting analysis of the heart tissues. Heart tissues were collected at 11 dpi from the 1-day-old C57BL/6 mice injected intraperitoneally with ZIKV strain PRVABC59 (10^6^ PFU/mouse) or PBS (*n* = 3/group). Homogenized heart tissues were subjected to western blot assays for Cx43, Tubulin, and ZIKV NS3 detection. (**B**) Western blotting analysis of the cardiomyocytes. Cardiomyocytes were isolated from the hearts of six 1-day-old mice and infected with ZIKV (MOI = 1) for 24 h. MG132 (5 or 10 µM), a ubiquitin-proteasome process inhibitor, was added to the cells 6 h before harvesting the cells. Whole-cell lysates were analyzed by western blot using anti-Cx43, anti-ZIKV NS3, and anti-tubulin antibodies. (**C**) RT-qPCR analysis of Cx43 mRNA levels. Relative Cx43 mRNA levels were measured in mock- or ZIKV-infected cardiomyocytes at 12, 24, and 48 hpi, normalized to GAPDH expression.

We previously demonstrated that ZIKV infection can activate the cellular ubiquitination pathway to degrade host proteins such as PCM1 (60). In our IHC assays, we observed a weaker Cx43 signal in the ZIKV-infected heart tissue compared to the PBS-treated control, raising the question of whether ZIKV infection downregulates Cx43 protein through ubiquitination-mediated degradation. To investigate this, we infected 1-day-old neonatal mice intraperitoneally with ZIKV (PRVABC59) at 10^4^ PFU per mouse, while control mice received the same volume of PBS. Each group has three mice, as shown in Fig. 6A. At 11 days post-infection (dpi), the mice were euthanized, and their hearts were isolated and homogenized for western blot assays using antibodies against Cx43, Tubulin, and NS3. The presence of a ~70 kDa NS3 protein band confirmed direct ZIKV infection in neonatal heart tissue. Additionally, Cx43 protein levels were consistently lower in ZIKV-infected mice compared to PBS-treated controls, suggesting ZIKV-mediated downregulation of Cx43.

To further examine this phenomenon, we isolated primary cardiomyocytes from the hearts of 1-day-old mice. Given that primary cardiomyocytes can only be cultured once, cells from six neonatal hearts were pooled and seeded into four wells. One well was mock-treated, while the remaining three were infected with ZIKV at an MOI of 1 for 24 h. To assess whether proteasomal degradation contributed to Cx43 reduction, we treated infected cells with MG132 (Sigma, CAS 133407-82-6), a ubiquitin-proteasome inhibitor, at 5 or 10 µM for 6 h prior to sample collection. Western blot analysis of whole-cell lysates (Fig. 6B) revealed that ZIKV infection significantly reduced Cx43 protein levels, while MG132 treatment effectively rescued Cx43 expression, suggesting that ZIKV-mediated Cx43 degradation occurs via the ubiquitin-proteasome pathway. To confirm that Cx43 protein reduction was not due to transcriptional downregulation, we performed the RT-qPCR assays to examine Cx43 mRNA levels in mock- or the ZIKV-infected cardiomyocytes at 12, 24, and 48 h post-infection (hpi). As shown in Fig. 6C, Cx43 mRNA levels remained unchanged between ZIKV-infected and mock-treated cells, indicating that ZIKV infection does not significantly affect Cx43 transcription. Collectively, these results demonstrate that ZIKV infection leads to the downregulation of Cx43 protein in neonatal mouse hearts and cardiomyocytes through ubiquitin-mediated proteasomal degradation, which may contribute to impaired cardiac function.

## DISCUSSION

Multiple viruses, including members of the Flaviviridae family such as Chikungunya virus and the Coronaviridae family such as SARS-CoV-2, have been implicated in viral myocarditis (61, 62). Our study expands on this concept by demonstrating that Zika virus (ZIKV), traditionally associated with neurodevelopmental damage, also directly infects and damages the neonatal heart. Importantly, this study uses immunocompetent neonatal mice—a model that mimics natural postnatal infection risk via breastfeeding or mosquito exposure—to investigate ZIKV-induced cardiac injury.

One of the key findings of our work is the clear evidence of ZIKV replication in the heart, leading to cardiac enlargement, elevated heart-weight-to-body-weight ratios, and electrocardiographic abnormalities. These structural and functional abnormalities coincide with high levels of viral RNA and biomarkers indicative of myocardial injury, including elevated cTnT, cTnI, LDH, CK, α-HBDH, and CK-MB in both heart tissue and serum. These biochemical indicators mirror profiles seen in acute myocardial infarction and support the hypothesis that ZIKV infection compromises cardiac integrity.

Inflammation emerged as a central pathogenic mechanism in ZIKV-induced cardiac dysfunction. Our cytokine profiling revealed significant upregulation of chemokines and cytokines known to mediate cardiac injury, including CCL2, CXCL9, and CXCL10. These molecules are clinically associated with heart failure and poor cardiovascular outcomes. Notably, CXCL10 has been implicated in both adult viral myocarditis and neuroinflammation during ZIKV infection, suggesting it may serve as a common mediator of ZIKV-induced tissue damage.

A novel mechanistic insight of our study is the disruption of connexin 43 (Cx43), a critical gap junction protein required for synchronous electrical conduction between cardiomyocytes (63, 64). We found that ZIKV infection not only altered the localization of Cx43 at intercalated discs but also significantly reduced its protein levels without altering mRNA expression. This suggests a post-translational degradation mechanism. Our *in vitro* assays using proteasome inhibitor MG132 confirmed that Cx43 degradation is mediated by the ubiquitin-proteasome system—an emerging theme in viral manipulation of host proteins. These findings mirror our earlier studies showing ZIKV-induced ubiquitination and degradation of PCM1, another structural protein critical for cellular architecture (60).

Masson’s trichrome staining and cleaved caspase-3 staining further revealed myocardial fibrosis and apoptosis, respectively. These structural changes likely contribute to the observed EKG abnormalities and cardiac dysfunction. Together, our data support a multifaceted model of ZIKV-induced cardiomyopathy: the virus directly infects the heart, disrupts organelle and cytoskeletal architecture, degrades key electrical conduction proteins, and triggers damaging inflammatory responses.

While congenital ZIKV infection has been well studied, our findings highlight the underappreciated risk of severe cardiac consequences following postnatal ZIKV infection in neonates. Importantly, our work suggests that cardiac involvement may contribute significantly to ZIKV-induced mortality, potentially in conjunction with or independent of neurological impairment. This could explain instances where microcephaly alone does not account for fatal outcomes.

In conclusion, this study establishes a robust neonatal mouse model to study ZIKV-induced cardiac pathogenesis. Future studies will focus on dissecting the molecular pathways linking inflammation, Cx43 degradation, and mitochondrial dysfunction in ZIKV-infected hearts. Therapeutically, our findings suggest potential for targeting host proteostasis (e.g., proteasome inhibition) and inflammatory mediators (e.g., CXCL10 antagonists) to mitigate virus-induced cardiac injury. Moreover, evaluating additional flaviviruses and ZIKV strains will help determine whether this cardiotropism is a shared feature among related pathogens.
